# Diversification by CofC and Control by CofD Govern Biosynthesis and Evolution of Coenzyme F_420_ and Its Derivative 3PG-F_420_

**DOI:** 10.1128/mbio.03501-21

**Published:** 2022-01-18

**Authors:** Mahmudul Hasan, Sabrina Schulze, Leona Berndt, Gottfried J. Palm, Daniel Braga, Ingrid Richter, Daniel Last, Michael Lammers, Gerald Lackner

**Affiliations:** a Junior Research Group Synthetic Microbiology, Leibniz-Institute for Natural Product Research and Infection Biology, Jena, Germany; b Institute of Biochemistry, Dep. Synthetic and Structural Biochemistry, Felix-Hausdorff-Str. 4, University of Greifswald, Greifswald, Germany; c Dept. Biomolecular Chemistry, Leibniz-Institute for Natural Product Research and Infection Biology, Jena, Germany; University of California, Irvine

**Keywords:** bacterial metabolism, biosynthesis, coenzyme, enzyme catalysis, substrate specificity, X-ray crystallography

## Abstract

Coenzyme F_420_ is a microbial redox cofactor that mediates diverse physiological functions and is increasingly used for biocatalytic applications. Recently, diversified biosynthetic routes to F_420_ and the discovery of a derivative, 3PG-F_420_, were reported. 3PG-F_420_ is formed via activation of 3-phospho-d-glycerate (3-PG) by CofC, but the structural basis of substrate binding, its evolution, as well as the role of CofD in substrate selection remained elusive. Here, we present a crystal structure of the 3-PG-activating CofC from *Mycetohabitans* sp. B3 and define amino acids governing substrate specificity. Site-directed mutagenesis enabled bidirectional switching of specificity and thereby revealed the short evolutionary trajectory to 3PG-F_420_ formation. Furthermore, CofC stabilized its product, thus confirming the structure of the unstable molecule and revealing its binding mode. The CofD enzyme was shown to significantly contribute to the selection of related intermediates to control the specificity of the combined biosynthetic CofC/D step. These results imply the need to change the design of combined CofC/D activity assays. Taken together, this work presents novel mechanistic and structural insights into 3PG-F_420_ biosynthesis and evolution and opens perspectives for the discovery and enhanced biotechnological production of coenzyme F_420_ derivatives in the future.

## INTRODUCTION

Organic cofactors like thiamin pyrophosphate (TPP), flavins (FAD, FMN), or nicotinamides (NAD^+^, NADP^+^) are small molecules other than amino acids that are required for the catalytic activity of enzymes (1). Knowledge about cofactor structure, function, and biosynthesis is therefore crucial to understand the biochemical and physiological processes their dependent enzymes are involved in and facilitates the biotechnological exploitation of these enzymes. Coenzyme F_420_ is a specialized redox cofactor that was so far mainly identified in archaea and some actinobacteria (2). In archaea, F_420_ is a key coenzyme of methanogenesis (3). In mycobacteria, F_420_ plays a vital role in respiration (4, 5), cell wall biosynthesis (6, 7), as well as the activation of medicinally relevant antimycobacterial (pro-)drugs. For instance, the novel anti-tubercular drug pretomanid is activated by Ddn, an F_420_-dependent nitroreductase (8, 9). In streptomycetes, F_420_H_2_ is used for reduction steps during the biosynthesis of antibiotics like thiopeptins (10), lanthipeptides (11), or oxytetracycline (12, 13). Increasing interest in F_420_ is also driven by the utilization of F_420_H_2_-dependent reductases in biocatalysis, for example, for asymmetric ene reductions (14–18).

Intriguingly, F_420_ also occurs in a few Gram-negative bacteria where it has been acquired most likely by horizontal transfer of its biosynthetic genes from actinobacteria (19, 20). Initial studies have revealed that F_420_ is indeed produced by some of these organisms but their physiological role remains unknown (20, 21). We have recently identified F_420_ biosynthetic genes in the genome of *Mycetohabitans* (synonym: *Paraburkholderia*) *rhizoxinica* (22), a symbiont that inhabits the hyphae and spores of the phytopathogenic mold Rhizopus microsporus (23–26). Surprisingly, we discovered that the symbiont produced a novel derivative of F_420_, which we termed 3PG-F_420_ (22). The cofactor activity of 3PG-F_420_ was comparable to classical F_420_ and could serve as a substitute for the latter in biocatalysis (22). Although this congener has not been described in any other organism, it could also be detected in the microbiota of a biogas production plant, thus demonstrating that it is not restricted to endofungal bacteria (22). The producers of 3PG-F_420_ in these habitats, however, are unknown. We hypothesize that analysis of organisms that have evolved a derivative of an otherwise conserved cofactor may also harbor unusual enzyme families that utilize this cofactor derivative. These enzymes could have novel activities or substrate specificities and are therefore of potential interest for biocatalysis.

**FIG 1:**
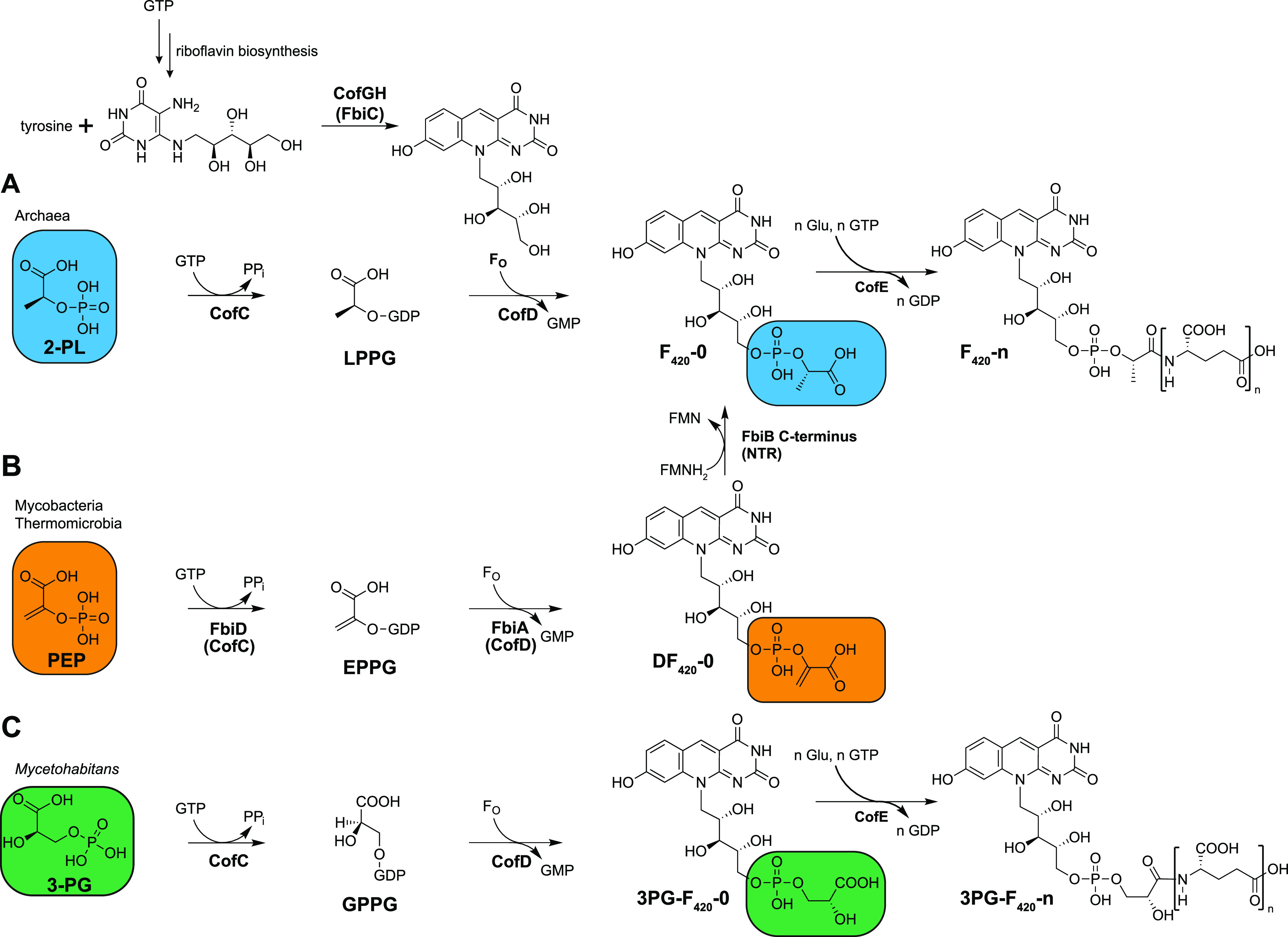
Biosynthesis of coenzyme F_420_ and biosynthetic steps performed by CofC and CofD during the formation of F_420_-species. (A) Formation of F_420_-0 from 2-PL as proposed for archaea. (B) Formation of the F_420_ precursor DF_420_, a pathway intermediate of F_420_ found in mycobacteria and Thermomicrobia. DF_420_ is further reduced to F_420_ by a nitroreductase (NTR)-like enzyme. (C) Biosynthesis of 3PG-F_420_-0 in *M. rhizoxinica* and related endofungal bacteria. 2-PL: 2-phospho-l-lactate, 3-PG: 3-phospho-d-glycerate, EPPG: enolpyruvyl-2-diphospho-5′-glutamate, FMN: flavin mononucleotide, F_O_: 7,8-didemethyl-8-hydroxy-5-deazariboflavin, GPPG: 3-(guanosine-5′-diphospho)-d-glycerate, Glu: glutamate, LPPG: l-lactyl-2-diphospho-5′-guanosine, NTR: nitroreductase, PEP: phosphoenolpyruvate, PP_i_: pyrophosphate.

The biosynthesis of 3PG-F_420_ (Fig. 1) is generally similar to the biosynthesis of classical F_420_ (27). The pathway starts with the formation of the redox-active core moiety 7,8-didemethyl-8-hydroxy-5-deazariboflavin (F_O_) from l-tyrosine and 5-amino-6-ribitylamino-uracil, a reactive metabolite of the flavin biosynthesis pathway. The F_O_ core is then elongated by a chemical group that can formally be described as 2-phospho-l-lactate (2-PL) before an oligoglutamate tail is added. The biosynthesis of the 2-PL moiety has been the subject of several studies. Seminal work on archaea suggested that it is directly formed from 2-phospho-l-lactate: Incubation of cell extracts of Methanosarcina thermophila or Methanocaldococcus jannaschii with F_O_, 2-PL, and GTP led to the formation of F_420_-0 (28). Biochemical assays with purified CofC and CofD finally corroborated the model that the guanylyltransferase CofC catalyzes the reaction of 2-PL and GTP to lactyl-2-phospho-guanosine (LPPG) (29), which is then passed on to CofD to transfer the activated 2-PL moiety onto the precursor F_O_. However, the unstable nature of LPPG has prevented confirmation of its structure by NMR or mass spectrometry so far. The last biosynthetic step leading to the mature coenzyme F_420_ is catalyzed by the F_420_:glutamyl ligase CofE (30), which is responsible for the addition of the *γ*-linked oligoglutamate moiety to the F_420_-0 core, thus forming F_420_-n, with n indicating the number of glutamate residues.

In mycobacteria, CofE is not a free-standing enzyme but constitutes the *N*-terminal domain of the FbiB protein (31). It was shown recently that mycobacteria utilize phosphoenolpyruvate (PEP), but not 2-PL, to form F_420_-0. Instead of LPPG, EPPG is formed, which is converted into dehydro-F_420_-0 (DF_420_-0) by the action of FbiA, the mycobacterial CofD homolog. DF_420_-0 is then reduced to classical F_420_-0 by the *C*-terminal domain of FbiB, which belongs to the nitroreductase superfamily (32). We have shown that a similar pathway is present in the thermophilic bacterium Thermomicrobium roseum and related species (33). The formation of 3PG-F_420_-0, however, does not require any reduction step. Instead, enzyme assays revealed that 3-phospho-d-glycerate (3-PG) is activated by CofC, presumably forming the short-lived intermediate 3-(guanosine-5′-diphospho)-d-glycerate (GPPG), which is further transferred to the F_O_ core by the action of CofD.

However, it remained elusive, which amino acid residues within the CofC protein conferred the specificity switch toward 3-PG and how genetic mutation might have led to the evolution of 3PG-F_420_ biosynthesis. Furthermore, the question persisted, why the CofC/CofD reaction only proceeds as a combined reaction and how reactive intermediates like LPPG are stabilized. Another open question concerned the role of 2-PL in the biosynthesis of F_420_ in archaea. While our previous data (22) matched seminal observations (29) of a substantial turnover of 2-PL by CofC enzymes of archaeal origin, other studies raised doubts that 2-PL is a genuine substrate of archaeal CofC homologs (32).

Here, we present a crystal structure of the 3-PG activating CofC from *Mycetohabitans* sp. B3 and revealed the amino acid residues governing 3-PG activation. By site-directed mutagenesis, we shed light on the evolution of 3PG-F_420_. Furthermore, we bring to attention that CofC strongly binds its product GPPG and collaborates closely with its partner CofD to control the flux of intermediates into the F_420_ biosynthesis pathway.

## RESULTS

### Assessment of substrate specificities of CofC enzymes from several organisms.

**FIG 2:**
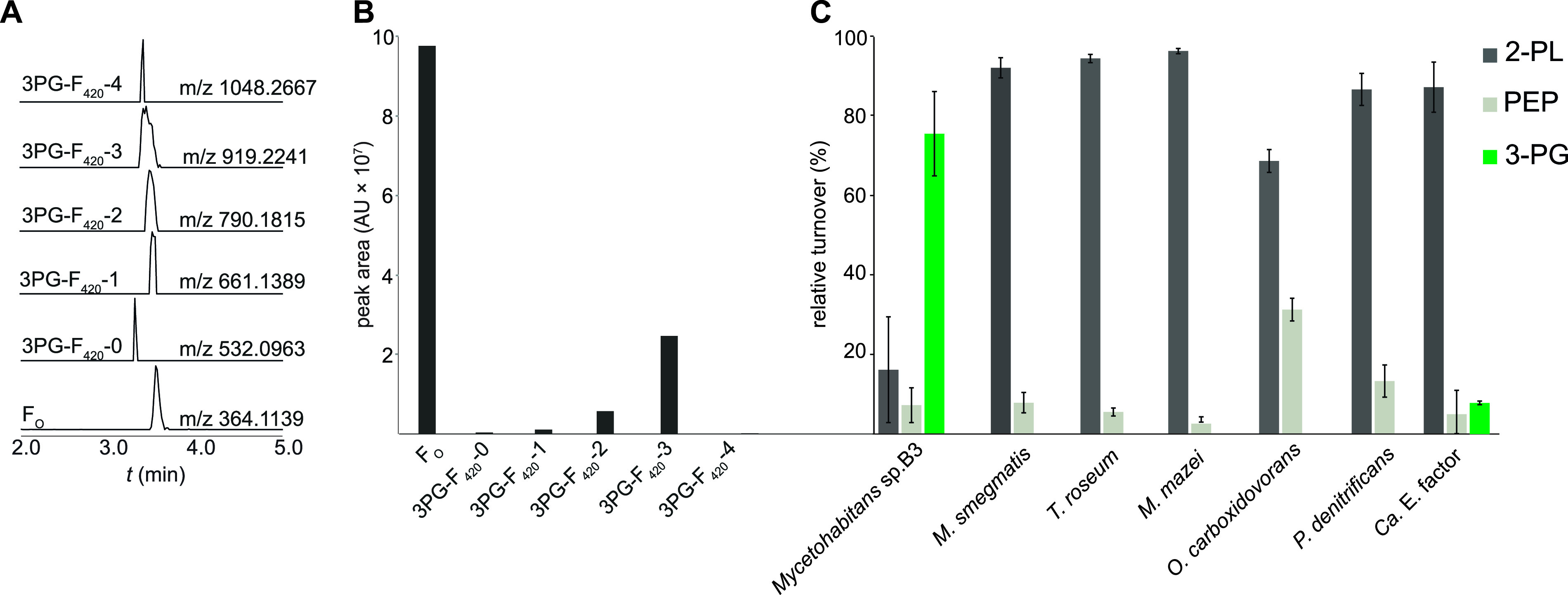
Production of 3PG-F_420_ and substrate specificity of CofC/FbiD enzymes. (A) Extracted ion chromatograms (XICs) of deazaflavin species extracted from *Mycetohabitans* sp. B3 (peaks are scaled to the same height). (B) Areas under the peaks (arbitrary units) depicted in panel A. (C) Substrate specificity assay of CofC/FbiD from various source organisms. The CofC of *Mycetohabitans* sp. B3 showed strong 3-PG activation while all other homologs preferred 2-PL. *Mjan*-CofD was combined with all CofC homologs to perform the CofC/D assay. Error bars represent the standard deviation of three biological replicates. Relative turnover of 3-PG, 2-PL, and PEP are reflected by the rate of 3PG-F_420_-0, F_420_-0, and DF_420_-0 formation, respectively.

To gain a better understanding of CofC substrate specificities, we set out to identify more homologs of CofC accepting 3-PG as a substrate. We reasoned that related bacteria, harboring CofC homologs highly similar to the *M. rhizoxinica* enzyme (*Mrhiz*-CofC), would have a similar substrate preference. Indeed, using LC-MS we detected 3PG-F_420_ (Fig. 2A/B) in cell extracts of *Mycetohabitans* sp. B3, a close relative of *M. rhizoxinica* that shares the same lifestyle as a symbiont of a phytopathogenic Rhizopus microsporus strain. Neither classical F_420_ nor DF_420_ was detectable.

Next, we produced CofC of *Mycetohabitans* sp. B3 (*Myc*B3-CofC, accession number KQH55_09515) as a hexahistidine fusion-protein in E. coli, purified the enzyme by Ni-NTA affinity chromatography, and investigated its activity in a combined CofC/D assay using CofD from Methanocaldococcus jannaschii (*Mjan*-CofD). When the substrates were provided in equal concentrations in a competitive assay, *Myc*B3-CofC (Fig. 2C) accepted 3-PG (65%), 2-PL (26.5%), and PEP (8.5%), a profile that was similar to the one obtained previously for the *Mrhiz*-CofC (22).

To obtain an overview of the substrate specificities of CofC we re-assessed CofC enzymes from well-studied F_420_ producing organisms such as Mycolicibacterium smegmatis, Thermomicrobium roseum, and Methanosarcina mazei (22) and assayed CofCs from further Gram-negative bacteria like Paracoccus denitrificans, Oligotropha carboxidovorans, as well as the uncultivable *Candidatus* (*Ca*.) Entotheonella factor TSY1 that is rich in genes encoding F_420_-dependent enzymes (21). For all CofC-related enzymes analyzed, 2-PL was used most efficiently from all substrates compared. We observed PEP turnover in the range of 3.5% to 30% (Fig. 2C). Generally, it can be concluded that CofC assays cannot discriminate whether 2-PL or PEP is the relevant substrate *in vivo*. The only CofC that accepted 3-PG to a certain extent (8%) was the enzyme from *Ca.* E. factor. However, compared to the *Mycetohabitans* enzyme there was no significant preference of 3-PG over PEP.

### Identification of 3-PG-binding residues of CofC.

**FIG 3:**
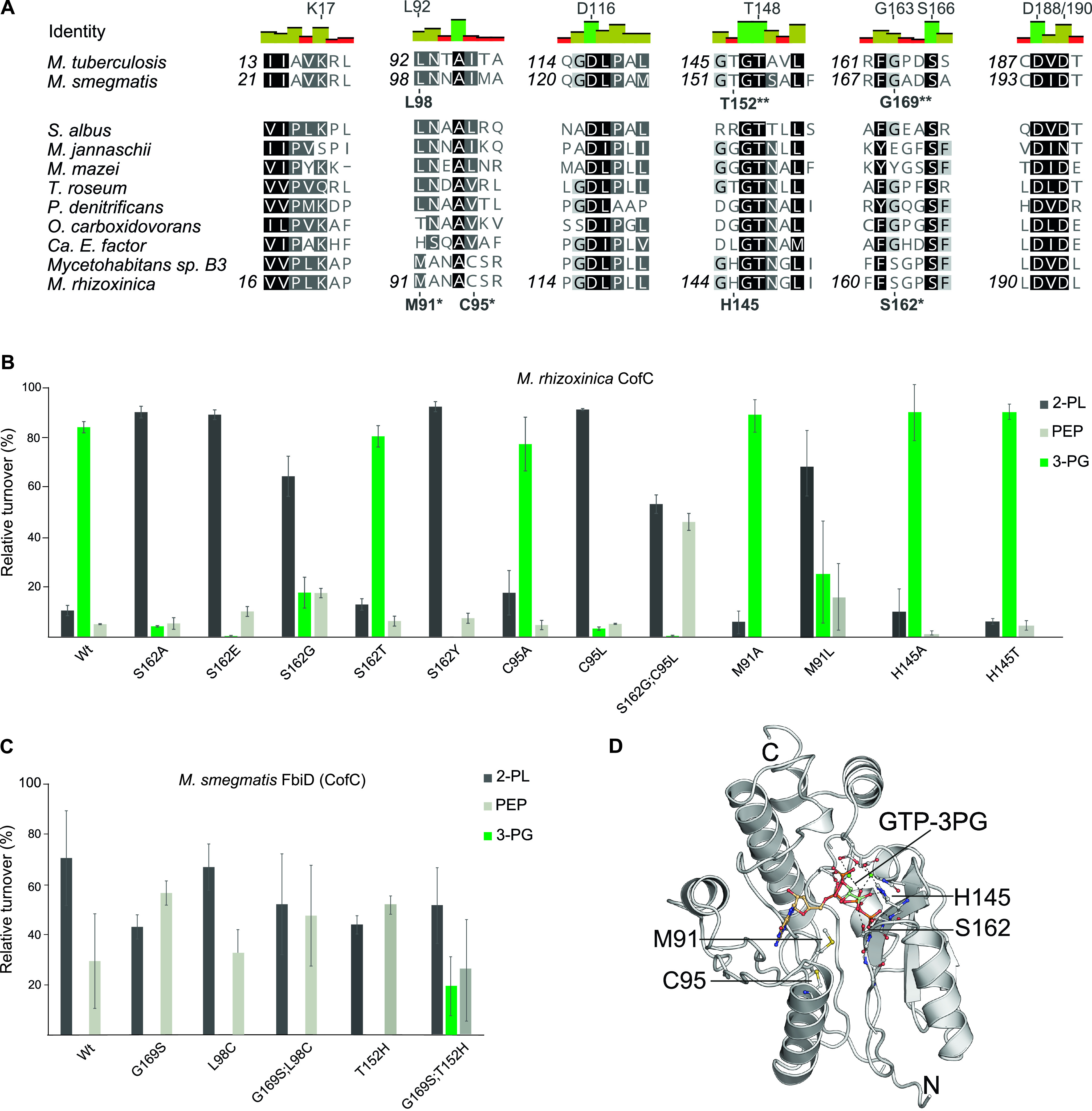
Residues determining substrate specificities of CofC homologs. (A) Multiple sequence alignment of CofC proteins from selected source organisms. Amino acids of *Mtb-*FbiD suggested previously to be involved in PEP binding residues (32) are indicated above the identity graph. Residues tested by mutagenesis are shown below sequences. Asterisks: crucial for 3-PG activation in *Mrhiz-*CofC, double asterisk: enabled 3-PG activation by *Msmeg*-FbiD. (B) Substrate specificity of *Mrhiz-*CofC after site-directed mutagenesis. Substitution of S162, C95, and M91 by residues occurring in 2-PL/PEP activating enzymes led to reduction or abolishment of 3-PG activation. (C) Substrate specificity of *Msmeg*-FbiD after site-directed mutagenesis. Gly169, Leu98, and Thr152 were exchanged by amino acids found in 3-PG activating homologs. While single mutations did not result in 3-PG activation, the combination G169S;T152H enabled 3-PG turnover. Error bars represent the standard deviation of three biological replicates. (D) Homology model of *Mrhiz*-CofC in complex with GTP (placed by molecular docking) and 3-PG (placed manually). For details, see Fig. S1 in the supplemental material.

Next, we aligned primary amino acid sequences of CofC homologs to identify the residues that might be responsible for the altered substrate preference (Fig. 3A). A crystal structure of FbiD from Mycobacterium tuberculosis (*Mtb*-FbiD) in complex with PEP (PDB: 6BWH) showed eight amino acid residues to be in close contact with PEP suggesting a role in conferring the substrate specificity (32). Three of them are aspartate residues (D116, D188, D190) that complex two Mg^2+^ ions which in turn interact with the phosphate group of PEP. The remaining residues were supposed to bind the PEP molecule via side chain atoms (K17, L92, S166) or backbone amino groups (T148 and G163). Although most of these residues were highly conserved, two alignment positions showed a deviation in those residues, namely, L92 and G163 of *Mtb*-FbiD. While L92 is replaced by methionine (M91), the residue corresponding to FbiD-G163 was replaced by serine (S162) in *Mrhiz*-CofC and *Myc*B3-CofC. Homology modeling further suggested H145 to be a potentially critical residue for 3-PG binding and C95 to be involved in the correct positioning of M91 (Fig. 3D, Fig. S1 in the supplemental material).

10.1128/mbio.03501-21.1 FIG S1 Homology model of *M. rhizoxinica* CofC in complex with GTP and 3-PG. (A) Electrostatics surface representation of a structural model of CofC in complex with GTP. GTP binds in a charged solvent-exposed cavity involving two Mg^2+^ ions. The electrostatic surface potential was calculated using the APBS. Red: negative charge; blue: positive charge. (B) Cartoon representation of the CofC•GTP-3PG model. The model shows the state of nucleophilic attack of the phosphate of 3-PG (P_3PG_) to the α-phosphate of GTP (distance between P_3PG_ oxygen and P of α-phosphate is less than the Van der Waals distance). GTP is bound by two Mg^2+^ ions neutralizing the negative charges and polarizing the P-O bond of the α-phosphate for the nucleophilic attack. The GTP and 3-PG are shown in ball-and-stick representation. The position of S162 and H145 contacting the 3-PG carboxylate and of M91 and C95 are shown. (C) Closeup of the proposed interaction of GTP and 3-PG with CofC shown in B. Mg^2+^ ion A (M_A_) is coordinated by D116, D193, and the α-/β-phosphates of GTP. Mg^2+^ ion B (M_B_) is coordinated by D191, D193, N141, H145, and the β-phosphate of GTP. Moreover, the octahedral coordination of the Mg^2+^ ions is most likely completed by water molecules which are not shown in this model. GTP γ-phosphate contacts main-chain amide nitrogens of S162 and G146. The P_3PG_ contacts M_A_, the carboxylate of 3-PG (C) is in hydrogen bond distance to the side chains of S162 and H145. Mutation of C95L affects the orientation of M91 interfering with the 3-PG hydroxyl group likely affecting 3-PG binding and allowing activity towards 2-PL. GTP is shown in orange, 3-PG in green. Download FIG S1, TIF file, 2.6 MB. Copyright © 2022 Hasan et al. 2022 Hasan et al. https://creativecommons.org/licenses/by/4.0/ This content is distributed under the terms of the Creative Commons Attribution 4.0 International license.

### Mutagenesis of CofC reveals S162 to be crucial for 3-PG activation.

To probe the role of the suggested residues, we performed site-directed mutagenesis in *Mrhiz*-CofC (Fig. 3B). Especially S162 turned out to be critical for 3-PG activation. While the least invasive mutation, S162T, retained most of the activity of wild-type CofC toward 3-PG, all other mutants of this residue preferentially turned over 2-PL and, to a lesser extent, PEP. This finding suggested that the hydroxy group present in S162 of WT and S162T might support the recruitment of 3-PG to the active site. M91L displayed reduced activation of 3-PG, while M91A was not impaired in 3-PG activation. Possibly, M91 controls the size of the substrate-binding pocket thus hindering (M91L) or facilitating (M91A) access of the larger substrate 3-PG to the active site. The C95A mutant approximately retained wild-type activity toward 3-PG, while C95L strongly reduced 3-PG activation. This was an indication that C95 might indeed affect the orientation of M91 and as a consequence 3-PG binding. Finally, the proposed interaction of 3-PG with H145 was not reflected in altered specificity profiles of H145A and H145T mutants of *Mrhiz*-CofC.

### Engineering M. smegmatis FbiD into a 3-PG activating enzyme.

Inspired by the finding that S162 of the *Mrhiz-*CofC is necessary for 3-PG activation we wondered if mutation of the corresponding residue G169 of FbiD to serine (Fig. 3C) could turn FbiD from M. smegmatis (*Msme*g-FbiD) into a 3-PG activating enzyme, thereby imitating the molecular processes underlying the evolution of 3PG-F_420_ biosynthesis. The G169S mutant, however, did not accept any 3-PG as substrate. We also mutated L98 to facilitate the entry of 3-PG into the active site. However, neither the single mutant L98C, nor the double mutant G169S;L98C enabled 3-PG binding. Based on the homology model we suspected a residue corresponding to H145 of the *Mrhiz*-CofC might facilitate 3-PG binding. Indeed, while the single mutant T152H did not show any significant effect, the double mutant G169S;T152H successfully turned over 3-PG (19.6%). The triple mutant (G169S;T152H;L98C) resulted in insoluble protein. Overall, these results showed that substrate specificity of *Msme*g-FbiD can be readily switched by changing only two residues and again supported a prime role of the critical serine residue for 3-PG recruitment.

### Structural insights into 3-PG activation by CofC.

**FIG 4:**
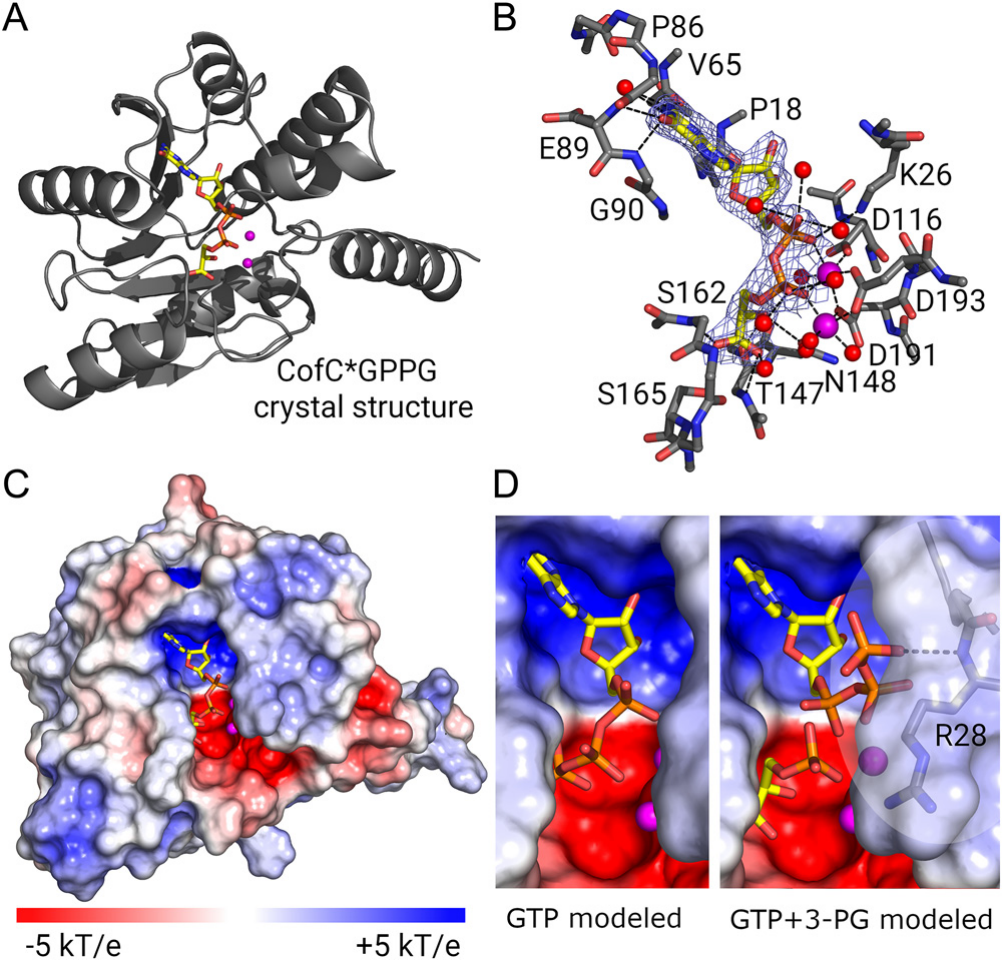
Structures of *Myc*B3-CofC in complex with GPPG (crystal structure) and educts (model). (A) Ribbon diagram of the crystal structure of CofC with its product GPPG. (B) Final electron density of GPPG (2F_o_F_c_ at 1.5 σ) and H-bonds for GPPG binding. Side chains are only shown for residues contacting the ligand. (C) Electrostatic surface representation of the protein shows a negatively charged deep pocket for GPPG. Two Mg^2+^-ions allow binding of the charged phosphates of GPPG. The electrostatic surface potential was calculated using APBS. Red: negative charge; blue: positive charge. (D) Possible educt conformations. GTP could position its α- and β-phosphates where GPPG binds to the Mg^2+^-ions (left side). With 3-PG binding in the same mode as the 3-PG moiety of GPPG the β- and γ-phosphates of GTP have to rotate out of the binding pocket, though (right side). The phosphate moiety of 3-PG is well poised for nucleophilic attack on the α-phosphate of GTP via a trigonal bipyramidal transition state. Figures were produced with PyMOL. A window around R28 is transparent to show the potential H-bond of GTP to the amide nitrogen. Electrostatic surface representations were made using the APBS electrostatics plugin in PyMOL. Carbon: yellow, oxygen: red, nitrogen: blue, magnesium: magenta.

After several attempts had failed to crystallize recombinant *Mrhiz*-CofC we turned to *Myc*B3-CofC that was more soluble despite only minor differences in the amino acid sequence (96,8% sequence identity). From diffraction data collected to 2.4 Å the crystal structure could be solved by molecular replacement with a model of two superimposed structures (*Mtb-* FbiD and Methanosarcina mazei CofC, *Mmaz*-CofC). The overall structure was similar to the known homologs with the core of the single-domain protein being a six-stranded mixed β-sheet (Fig. 4).

Intriguingly, after initial refinement both molecules in the asymmetric unit independently showed unambiguous difference density for GPPG, the reaction product of GTP with 3-PG, completely immersed into the active site pocket. Each building block of GPPG (i.e., guanine, ribose, phosphate, and glycerate) is bound by several interactions (Fig. 4B, Table S1). Guanine is distinguished from adenine by two H-bond donors to its oxygen (main-chain nitrogen of V65 and P86) and two H-bond acceptors to its amino group (main-chain carbonyl group of E89 and G90). The α- and β-phosphates are bound by two Mg^2+^ ions, which in turn are positioned by three aspartates (D116, D193, D191) similarly as had been shown for FbiD before (32). In the homologous structures the binding site for guanosine and ribose is almost completely conserved. Hence, GPPG is tightly bound, in fact, it turned out to be still quantitatively bound after purification of the protein from E. coli cell lysate, where both substrates were present.

10.1128/mbio.03501-21.4 TABLE S1 Ligand interactions of *Myc*B3-CofC with GPPG. Interactions of 3-GPPG with MycB3-CofC are given with their average distance (for chain A and B). Residues which are indirectly contacted via a water molecule or a magnesium ion are shown in parentheses. Analogous residues in *Mtb*-FbiD and *Mmaz*-CofC are listed if assignable. In this case, distances are only given if a structure with ligand is available (i.e. PEP in *Mtb*-FbiD). Download Table S1, DOCX file, 0.02 MB. Copyright © 2022 Hasan et al. 2022 Hasan et al. https://creativecommons.org/licenses/by/4.0/ This content is distributed under the terms of the Creative Commons Attribution 4.0 International license.

The 3-PG moiety is primarily bound by H-bonds with the side chain of S165 to the carboxy group, apparently a highly conserved binding site of the carboxylate of the C3-acid as known from *Mtb*-FbiD (32). More interactions with the carboxy group arise from H-bonds with main-chain nitrogen atoms of T147 and S162, the latter being critical for selective 3-PG activation. Although the mild effect of S162T suggested otherwise, S162 did not interact via its hydroxy group with the ligand. Furthermore, there are H-bonds from the main-chain carbonyl groups of T147 and N148 to the 2-hydroxy group of 3-PG. Surprisingly, these binding partners of the 3-PG hydroxy group are structurally very well conserved and thus not likely to be involved in discrimination between 3-PG and 2-PL/PEP. The residue corresponding to *Mtb*-FbiD-K17 (*Mrhiz*-CofC-K20) did not interact with the substrate however, instead, K26 has taken over its role.

Taken together, the majority of the residues forming the binding pocket residues of the PEP-binding FbiD (32) were also found to be involved in 3-PG binding. Although all C3-acids form the same hydrogen bonds, the carboxylate rotates by 36° about the axis through the carboxylate defined by the oxygens. This moves the phosphate by 2–3 Å. The hydrogen bonds for PEP have a more favorable geometry (average out-of-π-plane distortion 0.74 Å) than those for 3-PG (1.38 Å) but the phosphate group will not be positioned properly anymore to attack the α-phosphate of GTP. 3-PG with one more bond (carboxylate-C2-C3-O-P compared to carboxylate-C2-O-P in PEP or 2PL) can compensate for the new orientation of the carboxylate. The reason why the 3-PG adopts a new orientation is the main-chain rotation of S162, the CA and CB of which then squeeze the 3-PG into the productive conformation.

Based on the position of GPPG, the binding site for GTP is evident for the GMP moiety. Differential scanning fluorimetry (nano-DSF) measurements further corroborated the direct binding of GTP by CofC (K_D_ <20 μM) (see Text S1, Fig. S3, Table S5 in the supplemental material). The β-phosphate can either bind in the same position as the second phosphate of GPPG or point outward into the solvent close to R28. The latter conformation allows the second substrate 3-PG to bind like PEP in FbiD. GTP and 3-PG are then positioned well for the reaction, the nucleophilic attack of the 3-PG phosphate on the α-phosphate of GTP (Fig. 4D).

10.1128/mbio.03501-21.3 FIG S3 Determination of CofC*GTP dissociation constants. (A) The fit to the unfolded fraction of CofC (f_u_) ignoring the presence of GPPG. (B) Dissociation constants of CofC*GTP and CofC*GPPG. Possible combinations of K_diss,GTP_ and K_diss,GPPG_. Download FIG S3, EPS file, 1.4 MB. Copyright © 2022 Hasan et al. 2022 Hasan et al. https://creativecommons.org/licenses/by/4.0/ This content is distributed under the terms of the Creative Commons Attribution 4.0 International license.

10.1128/mbio.03501-21.8 TABLE S5 Determination of CofC*GTP dissociation constants. Unfolding constants for CofC and dissociation constants of CofC*GTP ignoring the presence of GPPG. Download Table S5, DOCX file, 0.02 MB. Copyright © 2022 Hasan et al. 2022 Hasan et al. https://creativecommons.org/licenses/by/4.0/ This content is distributed under the terms of the Creative Commons Attribution 4.0 International license.

10.1128/mbio.03501-21.9 TEXT S1 Nano-DSF measurements. Download Text S1, DOCX file, 0.02 MB. Copyright © 2022 Hasan et al. 2022 Hasan et al. https://creativecommons.org/licenses/by/4.0/ This content is distributed under the terms of the Creative Commons Attribution 4.0 International license.

### Identification of further 3-PG accepting enzymes.

After establishing that serine or threonine in the position corresponding to *Mrhiz-*CofC-S162 are linked to 3-PG formation, we hypothesized that the residue might be exploited as a diagnostic residue to identify further 3-PG activating enzymes. Going beyond highly related *Mycetohabitans* species, which can be expected to be 3PG-F_420_ producers, database searches revealed candidate proteins from as-yet uncultivated archaeal species (Fig. S2A in the supplemental material) that contained serine or threonine at the critical alignment position.

10.1128/mbio.03501-21.2 FIG S2 CofC homologs with diagnostic serine residue. (A) CofC primary protein sequence alignment including CofC from *Ca.* H. archaeon, Archaeon_GBE54128, and GBE_18477. Previously suggested substrate-binding amino acid residues from *M. tuberculosis* FbiD are marked with a black asterisk. Critical residues influencing 3-PG selection revealed in this study are highlighted by colored boxes. The diagnostic serine or threonine in alignment position 175 (red box) was predictive for 3-PG binding. (B) Substrate specificity of archaeal CofC enzymes harboring a diagnostic serine residue. *In-vitro* production of coenzyme F_420_-0 derivatives from CofCs (*Ca.* H. archaeon, Archaeon GBE54128, and Archaeon_GBE18477) and CofD (*M. jannaschii*) assays. Error bars represent the standard deviation from three biological replicates. Download FIG S2, EPS file, 2.2 MB. Copyright © 2022 Hasan et al. 2022 Hasan et al. https://creativecommons.org/licenses/by/4.0/ This content is distributed under the terms of the Creative Commons Attribution 4.0 International license.

Since their source organisms were not accessible, we obtained the coding sequences of three of these candidate enzymes as synthetic genes and tested their substrate specificities (Fig. S2B). Interestingly, all of those enzymes accepted 3-PG as substrates. The circumstance that 2-PL was the best substrate of all three enzymes does not rule out the possibility that these enzymes are involved in 3PG-F_420_ formation given the fact that 2-PL is the default case for many enzymes examined in our assay system even if PEP is the natural substrate. Notably, two of the enzymes tested did not accept PEP as a substrate, a rather unusual finding. In the absence of 2-PL, this profile would result in the production of 3PG-F_420_. Taken together, S162 represents a diagnostic residue correlated with specificity or tolerance of CofC toward 3-PG.

### Evolution of 3-PG accepting enzymes.

**FIG 5:**
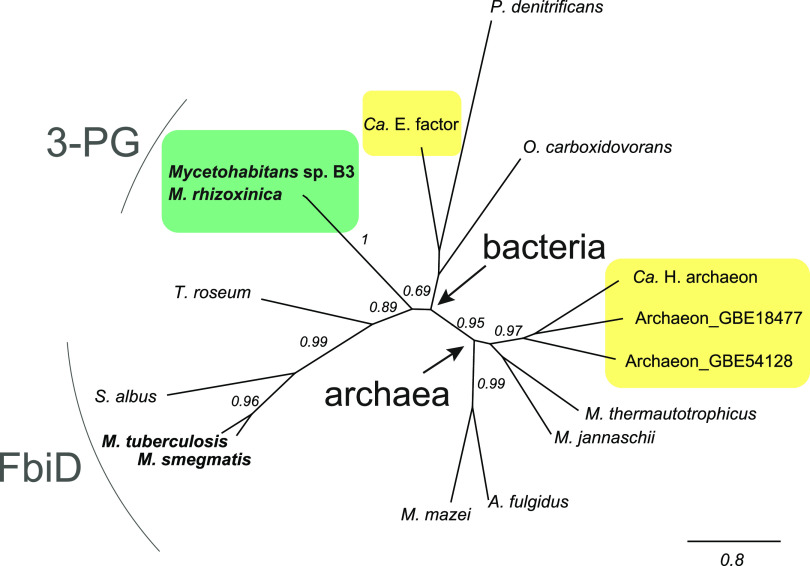
Phylogenetic tree of CofC/FbiD enzymes. The maximum likelihood method implemented in PhyML 3.0 was used to reconstruct the phylogeny. 3-PG activating enzymes are highlighted in green boxes, 3-PG tolerating enzymes in yellow boxes. Branch labels indicate SH-like support values. The scale bar represents substitutions per site. Accession numbers and source organisms of primary amino acid sequences are available in Table S3.

To answer the question how 3-PG accepting enzymes might have evolved, we constructed a phylogenetic tree of CofC enzymes examined in this study (Fig. 5). The *Mycetohabitans* CofC clade branched off early in the evolution of bacterial CofC enzymes and is neither closely related to nor derived from actinobacterial CofC/FbiD nor to other CofC enzymes found in Gram-negative bacteria. The archaeal 3-PG tolerating enzymes represent a monophyletic clade within the archaeal proteins. Taken together, we conclude that 3-PG preference evolved once in evolution while 3-PG tolerance originated at least twice from an ancestral 2-PL/PEP activating enzyme.

10.1128/mbio.03501-21.6 TABLE S3 Protein sequences of CofC/FbiD and CofD/FbiA homologs used in this study. Download Table S3, DOCX file, 0.02 MB. Copyright © 2022 Hasan et al. 2022 Hasan et al. https://creativecommons.org/licenses/by/4.0/ This content is distributed under the terms of the Creative Commons Attribution 4.0 International license.

### Role of CofD in substrate specificity of F_420_ side chain biosynthesis.

After gaining insights into the substrate specificity of CofC a few questions remained. For instance, in almost all CofC homologs tested, 2-PL was the preferred substrate. This finding contrasted previous results for *Mtb*-FbiD and CofC from *M. jannaschii* (*Mjan*-CofC), which was reported to accept exclusively PEP (32). Furthermore, the residual activity of *Mrhiz*-CofC and *Myc*B3-CofC toward PEP suggested the PEP-derived DF_420_ to be formed as a side product, while DF_420_ was not found in their source organisms (22). We therefore assumed that the choice of the CofD homolog used in the combined CofC/D assay might have an impact on the overall outcome of the assay.

**FIG 6:**
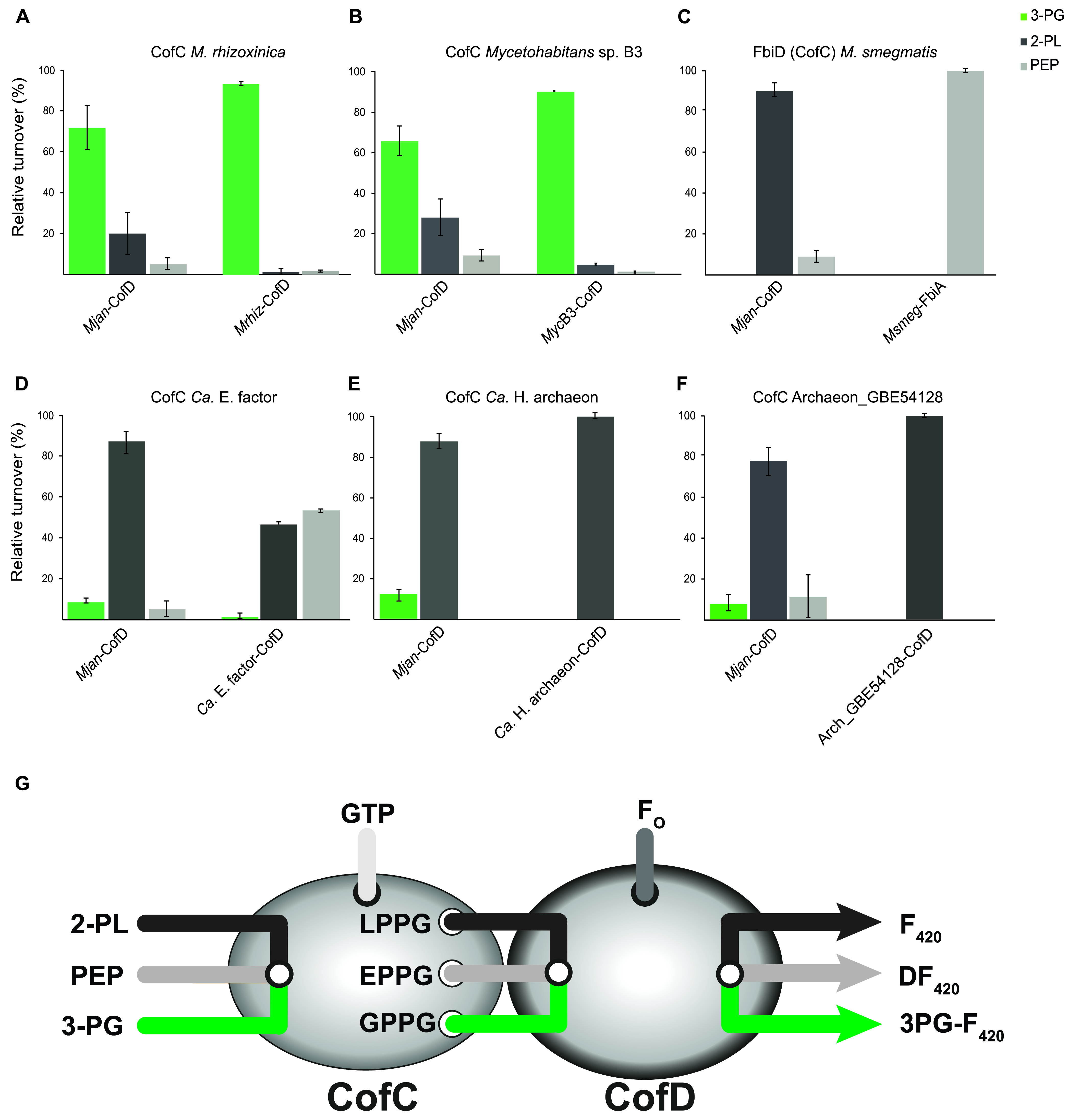
Influence of CofD/FbiA on the biosynthesis of F_420_-0 derivatives. (A) CofC from *M. rhizoxinica*, (B) CofC from *Mycetohabitans* sp. B3, and (C) FbiD from M. smegmatis, as well as (D) *Ca.* E. factor, (E) *Ca.* H. archaeon, and (F) Archaeon GBE54128 were tested with either *M. jannaschii* CofD (standard assay) or together with their cognate CofD enzyme from the same source organism. (G) Schematic drawing illustrating the channeling of the labile metabolites LPPG, EPPG, GPPG from CofC to CofD. While CofC selects primary substrates, CofD is able to select between these intermediates. For abbreviations see Fig. 1.

To test this hypothesis, we produced CofD homologs of several model species as hexahistidine-fusion proteins and performed CofC/D assays using several combinations of CofC and CofD. Strikingly, the choice of CofD homologs had a significant influence on the product spectrum of the CofC/D pair (Fig. 6). For instance, when *Mrhiz*-CofC and *Myc*B3-CofC were combined with their cognate CofD, the apparent substrate specificity shifted almost completely toward 3-PG. The PEP-and 2-PL-derived products were only produced in traces by the CofC/D reaction. Similarly, when *Msmeg*-FbiD was combined with its natural partner *Msmeg*-FbiA instead of the homolog encoded in *M. jannaschii* (*Mjan*-CofD), the apparent activity toward 2-PL was almost entirely abolished. Obviously, CofD homologs have the ability to select between the pathway intermediates LPPG (2-PL-derived), EPPG (PEP-derived), and GPPG (3-PG-derived). In bacterial systems, CofD/FbiA appears to favor the intermediate that is known to be relevant in their source organisms, i.e., EPPG for Mycobacteria or GPPG for *M. rhizoxinica/Mycetohabitans* sp. B3. The archaeal *Mjan*-CofD, however, appears to prefer its natural substrate LPPG but displays relaxed specificity toward EPPG and GPPG.

To clarify, whether CofCs that tolerate 3-PG to a certain extent could be involved in 3PG-F_420_ biosynthesis, we reassessed CofCs from *Ca.* E. factor, *Ca.* H. archaeon, and Archaeon GBE54128 together with their cognate CofDs (Fig. 6 D–F). However, the combined CofC/D pairs did not turn over any 3-PG. The archaeal CofC/D pairs were highly specific for 2-PL. Intriguingly, the *Ca.* E. factor CofC/D pair gained significantly higher preference for PEP than shown in the standard assay, suggesting that *Ca.* E. factor might produce F_420_ via DF_420_.

## DISCUSSION

### Structural basis of CofC specificity.

Extensive characterization of various CofC homologs, mutagenesis studies, and crystallography enabled us to spot residues responsible for the unusual substrate choice of CofC from *Mycetohabitans*. The crystal structure obtained from *Myc*B3-CofC revealed that most of the amino acid positions described for PEP-binding *Mtb*-FbiD play a role in 3-PG binding as well. However, rather than specific interactions with the free 2-hydroxy moiety, it is the conformation of S162 that forces the substrate into a position from which only the larger substrate 3-PG can undergo productive reaction of its phosphate group with GTP. The effect of M91 and C95 on substrate specificity shows that indirect influences on the overall conformation of the active site can be crucial for the correct positioning of the substrate. Nevertheless, the S162 residue proved as diagnostic residue correlated with tolerance against 3-PG and even enabled engineering of *Msmeg*-FbiD into a 3-PG activating enzyme. Notably, a bidirectional change of the substrate specificity, i.e., from 3-PG to 2-PL/PEP in *Mrhiz*-CofC as well as from 2-PL/PEP toward 3-PG in *Msmeg*-FbiD FbiD as described here is a rather exceptional achievement.

### Evolution and occurrence of 3PG-F_420_ in nature.

Interestingly, our mutagenesis study answers the question of how 3PG-F_420_ might have originated via mutation of 2-PL/PEP activating CofC on a molecular level. The phylogenetic tree suggests that 3-PG activating enzymes have evolved from an ancestral 2-PL/PEP activating CofC. Since DF_420_ is less stable than saturated forms, we suppose that the metabolic switching event has occurred to enable the formation of a stable F_420_-derivative in a metabolic background that lacked 2-PL or the DF_420_ reductase that is present in Actinobacteria (32) and Thermomicrobia (33). Here, we showed that the exchange of two amino acids in CofC/FbiD is mainly affecting substrate specificity and is thus sufficient to mimic this evolutionary process in the laboratory. Considering that 3PG-F_420_ was detectable in biogas-producing sludge (22), there must be microorganisms outside the monophylogenetic clade of endofungal bacteria (*Mycetohabitans*) that produce 3PG-F_420_. Efforts to isolate further 3PG-F_420_ producers remain ongoing.

### CofD influences substrate specificity of the CofC/D pair *in vivo* and *in vitro*.

Another important key finding of this study is that CofC and CofD together contribute to the substrate specificity of the combined reaction, where CofD of some species seems to represent a restrictive filter that acts after the more promiscuous CofC. This finding can explain the before-mentioned inconsistencies between results obtained *in vivo* and *in vitro*.

Even more importantly, we can now resolve the discrepancies concerning the results of CofC/D assays performed with CofC from archaea existing in the literature. Seminal work identified 2-PL to be a suitable substrate of archaeal CofC enzymes and thus proposed the original biosynthetic pathway to start from 2-PL (29). In contrast, a more recent study did not observe any turnover of 2-PL neither using the archaeal CofC from *M. jannaschii*, nor using FbiD from M. tuberculosis and suggested a biosynthetic route starting from PEP via EPPG, even for archaea (32). A follow-up study delivered further evidence supporting the biosynthetic route via DF_420_ in mycobacteria. The authors showed the formation of DF_420_ from PEP in cell extracts of M. smegmatis and could reveal strong binding of DF_420_ to the active site of FbiA (CofD) by X-ray crystallographic studies of the enzyme (34).

Our previous study (22), however, found 2-PL to be the best substrate of the *M. jannaschii* CofC enzyme, a result that we could reproduce here using CofC from *M. mazei*. It is also confusing that even the M. smegmatis FbiD tested in this study preferred 2-PL as substrate, again challenging the hypothesis that PEP is the preferred substrate of FbiD.

The solution to this perplexing situation comes with the herein defined influence of CofD/FbiA on the overall specificity of the CofC/D pair. CofD/FbiA selects between the unstable pathway intermediates LPPG, EPPG, and GPPG (27). According to our results, *Msmeg*-FbiA exclusively accepted EPPG to form DF_420_. Bashiri et al. used the closely related *Mtb*-FbiA to perform CofC/D assays (32). Since *Mjan*-CofC can accept PEP as a minor substrate when combined with its cognate CofD (22), the assay resulted exclusively in DF_420_-formation when combined with FbiA, thus erroneously suggesting that 2-PL was not accepted by CofC. Similarly, the activation of 2-PL by FbiD remained undetected when assays were carried out with FbiA as a partner enzyme. Conversely, it is plausible that the unexpectedly high turnover of 2-PL observed in all our assays performed with *Mjan*-CofD might be an artifact caused by the choice of a CofD homolog that might preferably turn over its natural substrate LPPG. For future studies toward the biosynthesis of novel F_420_ derivatives we, therefore, suggest that only a combination of CofC and its cognate CofD is suitable to reflect the *in vivo* situation. We also conclude that combining compatible CofC/D pairs will be beneficial for biotechnological production of F_420_.

### The combined CofC and CofD reaction.

The X-ray structure of *Myc*B3-CofC presented here included the reaction product GPPG, while the previous crystal structure of FbiD was obtained in the presence of PEP only (32). This is the first direct analytical evidence for the labile reaction product GPPG. So far, the existence of its congener LPPG was confirmed by chemical synthesis followed by successful turnover by CofD (35). The fact that GPPG remains tightly bound to the enzyme might point to a substrate-channeling mechanism where the GPPG molecule is directly transferred to CofD to avoid degradation of the labile intermediate in the absence of F_O_. Product inhibition could also explain why any attempt to measure the activity of CofC in the absence of CofD remained unsuccessful (27, 32) and why direct detection of GPPG or the related LPPG and EPPG from solution has failed so far.

The binding mode of GPPG also clearly revealed the GTP binding site of CofC. Notably, no evidence for GTP binding could be obtained experimentally for *Mtb*-FbiD and it was even speculated that GTP binding might require the presence of FbiA (32). This mechanism could be disproved for CofC of *M. rhizoxinia*.

### Conclusion.

Taken together, this study represents a significant advance in understanding the flexibility of substrate specificity in CofC homologs and offers a molecular model for the evolution of 3PG-F_420_. By direct detection of the instable reaction product GPPG via X-ray crystallography we gained insights into the structural basis of the combined CofC/D reaction. The demonstration that CofC and CofD cooperate closely to control the entry of central carbon metabolites into the biosynthetic route to F_420_ derivatives also solved an ongoing debate in the literature and thereby reestablished 2-PL as the most likely starting point of F_420_ biosynthesis in archaea. One important practical conclusion of this work is the suggestion that CofC/D assays should always be performed using homologs from the same source organism to better reflect the *in vivo* situation. Future perspectives are opened up to investigate the cooperation of biosynthetic enzymes on a molecular level and to exploit this knowledge gained here for enhanced biotechnological production of coenzyme F_420_ and potentially novel derivatives.

## MATERIALS AND METHODS

### Chemicals and microbial strains and metabolite extraction.

Chemicals and media components were purchased from Acros Organics, Sigma-Aldrich, Alfa Aesar, Carl Roth, and VWR. The E. coli strains BL21(DE3), LOBSTR-BL21 (Kerafast), ETH101 and Top10 were grown routinely in Lysogeny Broth (LB). Axenic *Mycetohabitans* sp. B3 (NCBI genome accession JAHLKN000000000) was grown in 50 ml MGY media (22) at 30°C at 110 rpm for 1 week. Cultures (OD_600_ = 2.5) were lyophilized, extracted with 10 ml ice-cold methanol, sonicated for 20 min and incubated at 250 rpm for 60 min. Cell debris was removed by centrifugation (4°C, 10 min, 10000 rpm) and the supernatant was filtered into a round-bottom flask. The extract was dried in a vacuum rotary evaporator at 40°C and re-dissolved in 1 ml LC-MS-grade water. Samples were analyzed using LC-MS as described before (22).

### Construction of expression vectors.

Unless stated otherwise, primers (Table S2A in the supplemental material) were designed using the software tool Geneious (36) and cloning was based on DNA recombination following the Fast Cloning protocol (37). The E. coli Top10 strain was used to propagate plasmids. PCRs were carried out using Q5 High-Fidelity polymerase (New England Biolabs) and oligomers used for amplifications listed in Table S1. Constructed plasmids (Table S2B) were confirmed by Sanger sequencing (Eurofins Genomics). CofC and CofD encoding plasmids (pMH04, 05, 10, 18, 19, 20, 43, 56, 57, 58, 59, 60, 89, 90 and 91) were purchased from BioCat as codon-optimized synthetic gene construct cloned into pET28a+ between BamHI and HindIII restriction sites. Plasmids pMH43 (pACYCDuet backbone), pMH59 (pET28), and pMH60 (pET28) were obtained by gene synthesis (BioCat), sequences are provided in the Supplemental Material (Text S2).

10.1128/mbio.03501-21.5 TABLE S2 Primers and plasmids used in this study. (A) Primer sequences (B) Plasmids used in this study. Download Table S2, DOCX file, 0.03 MB. Copyright © 2022 Hasan et al. 2022 Hasan et al. https://creativecommons.org/licenses/by/4.0/ This content is distributed under the terms of the Creative Commons Attribution 4.0 International license.

10.1128/mbio.03501-21.10 TEXT S2 Selected plasmid sequences. Download Text S2, DOCX file, 0.03 MB. Copyright © 2022 Hasan et al. 2022 Hasan et al. https://creativecommons.org/licenses/by/4.0/ This content is distributed under the terms of the Creative Commons Attribution 4.0 International license.

### Site-directed mutagenesis.

Putative substrate-binding residues of CofC were subjected to site-directed mutagenesis on DNA level using PCR (38). Amino acid numbering corresponds to the original residue position in the native CofC protein.

To mutate CofC from M. rhizoxinica (pFS03) at position 162 (S162), primer pairs oMH03/05, oMH05/06, oMH07/08, and oMH23/24 were used to generate S162E (resulting plasmid pMH22), S162Y (pMH23), S162G (pMH24), S162A (pMH25) and S162T (pMH26), respectively. Exchange of cysteine at position 95 (C95) was achieved using primer pairs oMH102/103 (C95L, pMH66) oMH104/105 (C95A, pMH67). The double mutant C95L;S162G (plasmid pMH68) was obtained from pMH24 by amplification with primer pair oMH106/107. Mutation of histidine at position 145 either to alanine (H145A) or threonine (H145T) was obtained using primer pairs oMH116/117 (pMH74) or oMH118/119 (pMH75). Methionine at position 91 was substituted with alanine (M91A) or leucine (M91L) to yield pMH76 and pMH77.

M. smegmatis *cofC* (pMH10) served as a template for substituting glycine residue in position 169 (G169). Primer pairs (oMH33/34) or (oMH37/38) or (oMH45/46) or oMH47/48 were used to obtain G169S (pMH32), G169Y (pMH33), G169A (pMH34) or G169E (pMH35), respectively. Primer pairs oMH108/109 were used to substitute leucine (L98) with cysteine (L98C, pMH70). The double mutants G169S; L98C and G169S;T152H were obtained from plasmid pMH32 using primer pair oMH110/111 and oMH124/125, yielding plasmid pMH69 and pMH80, respectively. The triple mutation was introduced in the pMH69 using oMH124/125 primers resulting in plasmid pMH81 (G169S;L98C;T152H).

### Heterologous protein production and purification.

Production condition and purification of all *N*-terminal hexahistidine (N-His_6_) tagged proteins (CofC and CofD) were similar as described before (22). Accession numbers of native CofC/FbiD and CofD/FbiA proteins are listed in Table S3. In short, chemical competent E. coli BL21(DE3) or LOBSTR-BL21 cells were transformed with individual CofC/CofD encoding plasmids and the respective antibiotic (kanamycin 50 μg/ml or chloramphenicol 25 μg/ml) was used to maintain selection pressure. Correct positive clones were grown overnight at 37°C and 180 rpm and used to inoculate fresh 100 ml cultures (1:100). Upon reaching late exponential growth phase (OD_600_ = 0.7), expression of the gene was induced by the addition of 1 mm IPTG and incubated (18°C, 180 rpm) following 18 h for protein production. After harvesting, cells were disrupted with pulsed sonication. The clear cell lysate was loaded onto a Ni-NTA affinity column to separate N-His_6_ tagged protein. Later on, the protein was eluted with a higher concentration of imidazole (500 mm) and re-buffered in a PD-10 column.

### Combined CofC/D assay.

Distinct derivatives of F_420_-0 were produced via biochemical reaction of purified CofC and CofD proteins in a combined assay (22, 29). *In vitro* reaction conditions were analogous to Braga et al. (22) and 50 μl reaction consisted of 100 mm HEPES buffer (pH 7.4), 2 mm GTP, 2 mm MgCl_2_, 0.14 nm Fo, 34 μm CofD, and 0.5 mm of substrates (3-phospho-d-glyceric acid, phosphoenolpyruvic acid, and 2-phospho-l-lactate). The reactions were initiated upon the addition of 26 μm CofC. Reactions were quenched with one volume of acetonitrile and formic acid (20%). Production of F_420_-0 derivatives was monitored in LC-MS. Technical set up, method, conditions for LC-MS analysis were similar as described before (22). Data analysis followed extraction of ion chromatograms (XICs), calculation of area under the curve (AUC), normalization of AUC, plotting area against time, and product formation was calculated for a linear time range (0 to 20 min). Quantification of relative product formation was determined from three biological replicates (*n* = 3) and plotted as bar charts. Standard deviations (SD) were used as error bars.

### CofC sequence alignment and phylogenetic tree inference.

Multiple protein sequences of CofC from different F_420_ producing organisms (Table S3) were retrieved from the NCBI database and primary sequences were aligned based on their predicted structure using Expresso (T-Coffee) (39). For phylogenetic tree inference as implemented in Geneious Prime (36), the MUSCLE algorithm (40) was used to align sequences and a maximum Likelihood tree was inferred using PhyML 3.0 (41) with the LG model for protein evolution and a gamma distribution of rates. Support values (Shimodaira-Hasegawa-like branch test) were computed and are shown above branches. Trees were visualized in Geneious Prime.

### Structural modeling.

The Phyre2.0 web portal was used to obtain a structural model for *M. rhizoxinica* CofC (PrCofC) (42). This enabled the identification of three enzymes (PDB: C3GX, PDB: 2I5E, PDB: 6BWH) that were used as a template for structural modeling resulting in models with 100% confidence in the fold. The enzyme FbiD from M. tuberculosis H37Rv (PDB: 6BWH) was used for further analyses. Initial structural alignment based on short fragment clustering of *M. rhizoxinica* CofC and FbiD was performed by program GESAMT (General Efficient Structural Alignment of Macromolecular Targets) from the CCP4i2 V1.0.2 program suite (43–45). This superimposed a total of 189 residues with an RMSD of 1.687 Å. FbiD binds to two Mg^2+^ ions that are important for catalysis and PEP binding (32). To place the Mg^2+^ ions in CofC, the CofC model was superposed on FbiD using residues surrounding the Mg^2+^ binding site. This model was used as a template for molecular docking of GTP into the CofC model using AutoDoc Vina. The input PDBQT files for AutoDoc Vina were generated with AutoDoc Tools V1.5.6 (46). The PRODRG server was used to generate the three-dimensional coordinates for GPPG from two-dimensional coordinates (47). The 3-PG was manually modeled in CofC•GTP using COOT (48). Representations of structures were prepared using PyMOL Molecular Graphics System (Schrödinger, LCC). The Adaptive Poisson-Boltzmann Solver (APBS) electrostatics plugin in PyMOL was used for the electrostatic surface representation (49).

### Crystallization and data collection.

*Mycetohabitans* B3 CofC was further purified on a size exclusion column (Superdex75, 16/600, Cytiva) and concentrated in 50 mm Tris pH 7.4, 100 mm NaCl, 5 mm MgCl_2_, 2 mm mercaptoethanol (SEC buffer) to 8.2 mg/ml. Sitting drop crystallization trials were set up with screens Wizard I and II (Rigaku), PEG/Ion (Hampton Research), and JBScreen (Jena Bioscience) using 0.3 μl protein solution and 0.3 μl reservoir. Crystals appeared after 2 weeks with reservoir 10% PEG 3000, 200 mm MgCl_2_, 100 mm sodium cacodylate pH 6.5. After briefly soaking in a reservoir with 20% glucose added crystals were cryocooled in liquid nitrogen. Diffraction data were collected at BESSY, beamline 14.1. Data collection parameters are given in Table S4 in the supplemental material. Data sets were processed with XDSAPP (50).

10.1128/mbio.03501-21.7 TABLE S4 Statistics of the X-ray diffraction data collection and structure refinement. Download Table S4, DOCX file, 0.02 MB. Copyright © 2022 Hasan et al. 2022 Hasan et al. https://creativecommons.org/licenses/by/4.0/ This content is distributed under the terms of the Creative Commons Attribution 4.0 International license.

### Structure solution and refinement.

Programs used for this part were all used as provided by CCP4 (45). Sequence search against the PDB revealed structures of two related proteins, FbiD from Mycobacterium tuberculosis and CofC from Methanosarcina mazei with sequence identities of 30%. Molecular replacement by PHASER with 6BWG (*Mtb*-FbiD) or 2I5E (*Mmaz*-CofC) did not solve in the automatic mode, an assembly of both structures superimposed and truncated (residues 9–82, 91-173, 178-211 of monomer A of 6BWG and aligned residues of monomer A of 2IE5) solved the phase problem with an LLG of 233. The 6BWG structure was used as starting model for replacing the sequence with the *Mycetohabitans* sequence, one round of automatic model building with BUCANEER (51) and iterative rounds of refinement with REFMAC5 (52) and manual model building with COOT (48) completed the two protein chains.

Unambiguous water molecules were added when R_free_ reached 0.315 and a GDP moiety with two Mg^2+^ ions was built into the difference density in the active site. Pertaining difference density was connected to the β-phosphate suggesting a covalently bound PEP or 3-PG. Only the latter refined without residual difference density above ±2σ. For final refinement, non-crystallographic symmetry was not used. TLS refinement was applied with one group per monomer. Refinement statistics are given in Table S4.

The C3-acid substrates are fixed in the active site by three H-bonds of their carboxylate group. For H-bonds to π-systems (peptide bond, carboxylate group) the partner should lie in the same plane. To characterize deviations from favorable H-bonding geometry we calculated the average distance of the partner to the π-plane.

### Data deposition.

The crystal structure of CofC from *Mycetohabitans* sp. B3 was deposited at the protein database PDB (PDB code: 7P97).
