# Supplementary material for: Diversification by CofC and Control by CofD Govern Biosynthesis and Evolution of Coenzyme F420 and Its Derivative 3PG-F420
Source: mBio. 2022 Jan 18;13(1):e03501-21. doi: 10.1128/mbio.03501-21 (PMC8764529; doi:10.1128/mbio.03501-21)
Supplement: TABLE S1 [file mbio.03501-21-st001.docx]

**Table S1:** **Ligand interactions of *Myc*B3-CofC with GPPG.**

Interactions of 3-GPPG with MycB3-CofC are given with their average distance (for chain A and B). Residues which are indirectly contacted via a water molecule or a magnesium ion are shown in parentheses. Analogous residues in *Mtb*-FbiD and *Mmaz*-CofC are listed if assignable. In this case, distances are only given if a structure with ligand is available (i.e. PEP in *Mtb*-FbiD).

| **Ligand atom** | ***Myc*B3-CofC with GPPG** | ***Mtb*-FbiD with PEP** | ***Mmaz*-CofC** |
| --- | --- | --- | --- |
| Guanine C6-O | E89N 3.01  G90N 2.79 | na  na | na  na |
| Guanine C2-NH_2_ | V65O 2.89  na | I62O  D82OD2 | L51O  na |
| Guanine N1 | HOH (P86O, E89OE1) 2.79 | na | na (D69O) |
| Ribose O2’ | CL 3.02  K20N 3.06 | na  K17N | na  K8N |
| Ribose O3’ | P18O 2.50  G115N 3.07 | A15O  G115N | P6O  A93N |
| Pα oxygen | K26NZ 3.15  MgA (D116OD2, D191OD2, D193OD1) 2.19 | K23NZ  MgA (D116OD2, D188OD2, D190OD1) | K13NZ  na (D94OD2, D168OD2, D170OD1) |
| Pβ oxygen | MgA (D116OD2, D191OD2, D193OD1) 2.18  MgB (D191OD2, D193OD2) 1.97 | MgA (D116OD2, D188OD2, D190OD1) 2.20  MgB (D188OD2, D190OD2) 2.15 | na (D94OD2, D168OD2, D170OD1)  na (D168OD2, D170OD2) |
| 3-PG 2-OH | T147O, 2.86  N148OD1, 2.99 | T148O no H-bond  no H-bond | T123O no H-bond  N124OD1 no H-bond |
| 3-PG COO^-^ | HOH (MgB) 2.72  T147N 2.98  S165OG 2.77  S162N 2.69 | HOH (MgB) 3.03  T148N 2.85  S166OG 2.59  G163N 2.70 | na  T123N  S142OG  T139N |

na: no analogous residue (or water molecule or ion) assignable in this structure
