# Supplementary material for: Diversification by CofC and Control by CofD Govern Biosynthesis and Evolution of Coenzyme F420 and Its Derivative 3PG-F420
Source: mBio. 2022 Jan 18;13(1):e03501-21. doi: 10.1128/mbio.03501-21 (PMC8764529; doi:10.1128/mbio.03501-21)
Supplement: TABLE S5 [file mbio.03501-21-st005.docx]

**Table S5:** **Determination of CofC*GTP dissociation constants.** Unfolding constants for CofC and dissociation constants of CofC*GTP ignoring the presence of GPPG.

| **T in °C** | 32 | 34 | 36 | 38 | 40 | 42 |
| --- | --- | --- | --- | --- | --- | --- |
| **K_u_** | 0.11 | 0.23 | 0.53 | 1.35 | 3.79 | 11.58 |
| **K_diss,GTP_ in μM** | 9.2 | 13.6 | 20.1 | 27.6 | 30.7 | 31.2 |
