# Supplementary material for: Diversification by CofC and Control by CofD Govern Biosynthesis and Evolution of Coenzyme F420 and Its Derivative 3PG-F420
Source: mBio. 2022 Jan 18;13(1):e03501-21. doi: 10.1128/mbio.03501-21 (PMC8764529; doi:10.1128/mbio.03501-21)
Supplement: TEXT S1 [file mbio.03501-21-s0001.docx]

# Supplementary Text 1

# Nano-DSF measurements

For differential scanning fluorimetry (DSF), CofC was diluted with SEC buffer (50 mM Tris-HCl, pH 7.4, 100 mM NaCl, 5 mM MgCl_2_, 2 mM β-mercaptoethanol) and a series of different GTP concentrations ([GTP]_final_ = 0 μM, 1 μM, 2 μM, 4 μM, 8 μM, 10 μM, 15 μM, 25 μM, 50 μM and 240 μM) to a concentration of 8 μM in a total volume of 20 μl. The samples were incubated for 10 min on ice before filled into glass capillaries (Prometheus^TM^ NT.48, Cat# PR-C002). Protein unfolding and intrinsic fluorescence (λ_ex_ = 285 nm, λ_ex_ = 330 nm) was measured using the Prometheus^TM^ NT.48 (NanoTemper) in the temperature range of 15 – 90 ˚C with a heating rate of 1 ˚C/min. Thermodynamic parameters (T_m_, ΔH_denaturation_ and Δc_p_) were calculated similar to Bai (1). Δc_p_ was fixed at 14235 Jmol^-1^K^-1^, fluorescence was normalized at 20 ˚C. Bai assumed only the unfolding reaction CofC_folded_ ⇌ CofC_unfolded_ and the binding reaction CofC_folded_ + GTP ⇌ CofC*GTP. Based on this framework, unfolded protein fraction can be fitted to obtain unfolding and dissociation constants (**Figure S3A, Table S5**).

From the crystal structure it is obvious, though, that the starting material is CofC*GPPG and not CofC_folded_. We have thus added the binding reaction CofC_folded_ + GPPG ⇌ CofC*GPPG with [GPPG]_total_ = [GPPG]_free_ + [CofC*GPPG] = 8 μM. This requires a second dissociation constant K_diss,GPPG_. Both dissociation constants cannot be fitted independently, but the values are correlated as shown in **Figure S3B**. The purified CofC*GPPG complex suggests that K_diss,GPPG_ is at most in the low μM range. In the measured temperature range GTP then binds ca. 10 times weaker than GPPG.

**References**

1. Bai, N., Roder, H., Dickson, A., and Karanicolas, J. (2019) Isothermal analysis of ThermoFluor data can readily provide quantitative binding affinities. *Sci Rep* **9**
