# Supplementary material for: Diversification by CofC and Control by CofD Govern Biosynthesis and Evolution of Coenzyme F420 and Its Derivative 3PG-F420
Source: mBio. 2022 Jan 18;13(1):e03501-21. doi: 10.1128/mbio.03501-21 (PMC8764529; doi:10.1128/mbio.03501-21)
Supplement: TABLE S3 [file mbio.03501-21-st003.docx]

**Table S3. Protein sequences of CofC/FbiD and CofD/FbiA homologs used in this study.**

| **Abbreviation** | **Accession number** | **Organism** | **Length(aa)** |
| --- | --- | --- | --- |
| **CofC/FbiD** |  |  |  |
|  | WP_010879553.1 | *Archaeoglobus fulgidus* DSM 8774 | 206 |
| Archaeon_GBE18477 | BMS3Abin16_01081 | Archaeon BMS3Abin16 | 214 |
| Archaeon_GBE54128 | BMS3Bbin15_00279 | Archaeon BMS3Bbin15 | 207 |
|  | ETW97754.1 | *Candidatus* Entotheonella factor TSY1 | 226 |
| *Ca.* H. archaeon | RLG60259.1 | *Candidatus* Hydrothermarchaeota archaeon | 207 |
| *Mjan-*CofC | WP_064496647.1 | *Methanocaldococcus jannaschii* DSM 2661 | 218 |
| *Mmaz*-CofC | WP_011034415.1 | *Methanosarcina mazei* Go1 | 208 |
| *Mrhiz*-CofC | WP_041753721.1 | *Mycetohabitans rhizoxinica* HKI 454 | 216 |
| *Msmeg*-FbiD | YP_886732.1 | *Mycobacterium smegmatis* str. MC2 155 | 221 |
|  | WP_010876252.1 | *Methanothermobacter thermautotrophicus* str. Delta H | 223 |
| *Mtb*-FbiD | P9WP83.1 | *Mycobacterium tuberculosis* H37Rv | 214 |
| *Myc*B3-CofC | KQH55_09515 | *Mycetohabitans* sp. B3 | 216 |
|  | WP_012562171.1 | *Oligotropha carboxidovorans* OM5 | 237 |
|  | WP_104493776.1 | *Paracoccus denitrificans* | 216 |
|  | WP_040247853.1 | *Streptomyces albus* | 211 |
|  | WP_012642646.1 | *Thermomicrobium roseum* DSM 5159 | 200 |
|  |  |  |  |
| **CofD/FbiA** |  |  |  |
| *Mjan*-CofD | WP_010870769.1 | *Methanocaldococcus jannaschii* DSM 2661 | 311 |
| *Mrhiz*-CofD | WP_013435881.1 | *Mycetohabitans rhizoxinica* HKI 454 | 328 |
| *Msmeg*-FbiA | WP_011727938.1 | *Mycobacterium smegmatis* str. MC2 155 | 327 |
| *Myc*B3-CofD | KQH55_09505 | *Mycetohabitans* sp. B3 | 323 |
| *Ca.* E. factor-CofD | ETW97753.1 | *Candidatus* Entotheonella factor TSY1 | 310 |
| *Ca.* H. archeon-CofD | RLG59732.1 (modified start codon) | *Candidatus* Hydrothermarchaeota archaeon | 308 |
| Arc_GEB54128-CofD | GBE54311.1 | Archaeon BMS3Bbin15 | 309 |
