# Supplementary material for: Diversification by CofC and Control by CofD Govern Biosynthesis and Evolution of Coenzyme F420 and Its Derivative 3PG-F420
Source: mBio. 2022 Jan 18;13(1):e03501-21. doi: 10.1128/mbio.03501-21 (PMC8764529; doi:10.1128/mbio.03501-21)
Supplement: TABLE S2 [file mbio.03501-21-st002.docx]

**Table S2: Primers and plasmids used in this study. (A)** Primer sequences **(B)** Plasmids used in this study.

**A**

| Name | 5`- 3` sequence |
| --- | --- |
| oMH03 | GTTTTTCGAAGGGCCAAGCTTTTCCGCTCATCAAAACGCC |
| oMH04 | CTTGGCCCTTCGAAAAACAACGGAATAGCGCCAGGTCG |
| oMH05 | GTTTTTCTATGGGCCAAGCTTTTCCGCTCATCAAAACGCC |
| oMH06 | CTTGGCCCATAGAAAAACAACGGAATAGCGCCAGGTCG |
| oMH07 | GTTTTTCGGCGGGCCAAGCTTTTCCGCTCATCAAAACGCC |
| oMH08 | CTTGGCCCGCCGAAAAACAACGGAATAGCGCCAGGTCG |
| oMH09 | GTTTTTCGCGGGGCCAAGCTTTTCCGCTCATCAAAACGCC |
| oMH10 | TTGGCCCCGCGAAAAACAACGGAATAGCGCCAGGTCG |
| oMH11 | GATCAAATATAGCGGCTTCAGCTTTCTGAAACATCTG |
| oMH12 | GCTGAAGCCGCTATATTTGATCTCGATCAGATCTTTGCTTTTC |
| oMH13 | GATCAAATATGGCGGCTTCAGCTTTCTGAAACATCTG |
| oMH14 | GCTGAAGCCGCCATATTTGATCTCGATCAGATCTTTGCTTTTC |
| oMH15 | GATCAAATATGCGGGCTTCAGCTTTCTGAAACATCTG |
| oMH16 | GCTGAAGCCCGCATATTTGATCTCGATCAGATCTTTGCTTTTC |
| oMH17 | GATCAAATATTATGGCTTCAGCTTTCTGAAACATCTG |
| oMH18 | GCTGAAGCCATAATATTTGATCTCGATCAGATCTTTGCTTTTC |
| oMH25 | GCTGGCCTTTGCGCATGATAGTTTTCAGCGTCATCTG |
| oMH26 | TATCATGCGCAAAGGCCAGCGGCAGACAATCCGGCGGGC |
| oMH27 | GCTGGCCTTTTATCATGATAGTTTTCAGCGTCATCTGAG |
| oMH28 | AAAACTATCATGATAAAAGGCCAGCGGCAGACAATCCGGC |
| oMH29 | GCTGGCCTTTGAGCATGATAGTTTTCAGCGTCATCTGAG |
| oMH30 | AACTATCATGCTCAAAGGCCAGCGGCAGACAATCCGGC |
| oMH31 | GCTGGCCTTTAGCCATGATAGTTTTCAGCGTCATCTGAGC |
| oMH32 | AACTATCATGGCTAAAGGCCAGCGGCAGACAATCCGGC |
| oMH33 | GCGCTTTAGCGCAGATAGTGCAACCCATCATCGCCATAG |
| oMH34 | CACTATCTGCGCTAAAGCGCGGCTGCAGCGGAACACCAA |
| oMH37 | GCCGCGCTTTTATGCAGATAGTGCAACCCATCATCGCCA |
| oMH38 | CACTATCTGCATAAAAGCGCGGCTGCAGCGGAACACCAA |
| oMH45 | GCTTTGCAGCAGATAGTGCAACCCATCATCGCCATA |
| oMH46 | TATCTGCTGCAAAGCGCGGCTGCAGCGGAACA |
| oMH47 | GCGCTTTGAGGCAGATAGTGCAACCCATCATCGCCATA |
| oMH48 | TATCTGCCTCAAAGCGCGGCTGCAGCGGAA |
| oMH23 | TTGTTTTTCACCGGGCCAAGCTTTTCCGCTCATCAAAACG |
| oMH24 | AGCTTGGCCCGGTGAAAAACAACGGAATAGCGCCAGGT |
| oMH102 | GAATGCCCTGTCGCGAGCGATGGCTCA |
| oMH103 | TCGCGACAGGGCATTCGCCATGCCTTC |
| oMH104 | GAATGCCGCGTCGCGAGCGATGGCT |
| oMH105 | CTCGCGACGCGGCATTCGCCATGCCTT |
| oMH106 | GAATGCCCTGTCGCGAGCGATGGCTCAT |
| oMH107 | CTCGCGACAGGGCATTCGCCATGCCTT |
| oMH108 | TAATCCGTGTAATAATGCCATTATGGCAGCAG |
| oMH109 | GCATTATTACACGGATTATGATGACCTTCAG |
| oMH110 | ATAATCCGTGTAATAATGCCATTATGGCAGC |
| oMH111 | GCATTATTACACGGATTATGATGACCTTCAGG |
| oMH124 | GCCATGGTCATGGTACCAGTGCACTGTTTGCATTT |
| oMH125 | CTGGTACCATGACCATGGCGATCGCCAACAAA |

**B**

| Name | Construct | Reference |
| --- | --- | --- |
| pMH03 | N-His_6_-CofC from *M. jannaschii* | Braga et al. 2020 (1) |
| pMH04 | N-His_6_-CofC from *Ca*. E. factor | This study |
| pMH05 | N-His_6_-CofC from *T. roseum* | This study |
| pMH10 | N-His_6_-CofC from *M. smegmatis* | This study |
| pMH18 | N-His_6_-CofC from *M. mazei* | This study |
| pMH19 | N-His_6_-CofC from *O. carboxidovorans* | This study |
| pMH20 | N-His_6_-CofC from *P. denitrificans* | This study |
| pMH22 | N-His_6_-CofC-S162E from *M. rhizoxinica* | This study |
| pMH23 | N-His_6_-CofC-S162Y from *M. rhizoxinica* | This study |
| pMH24 | N-His_6_-CofC-S162G from *M. rhizoxinica* | This study |
| pMH25 | N-His_6_-CofC-S162A from *M. rhizoxinica* | This study |
| pMH26 | N-His_6_-CofC-S162T from *M. rhizoxinica* | This study |
| pMH32 | N-His_6_-CofC-G169S from *M. smegmatis* | This study |
| pMH43 | N-His-CofC from *Mycetohabitans* sp. B3 | This study |
| pMH56 | N-His-CofC from *Ca.* H. archaeon | This study |
| pMH57 | N-His-CofC from Archaeon_GBE54128 | This study |
| pMH58 | N-His-CofC from Archaeon_GBE18477 | This study |
| pMH59 | N-His_6_-CofD from *M. smegmatis* | This study |
| pMH60 | N-His_6_-CofD from *Mycetohabitans* sp. B3 | This study |
| pMH66 | N-His_6_-CofC-C95L from *M. rhizoxinica* | This study |
| pMH67 | N-His_6_-CofC-C95A from *M. rhizoxinica* | This study |
| pMH68 | N-His_6_-CofC-S162G;C95L from *M. rhizoxinica* | This study |
| pMH69 | N-His_6_-CofC-G169S;L98C from *M. smegmatis* | This study |
| pMH70 | N-His_6_-CofC-L98C from *M. smegmatis* | This study |
| pMH74 | N-His_6_-CofC- H145A from M*. rhizoxinica* | This study |
| pMH75 | N-His_6_-CofC- H145T from M*. rhizoxinica* | This study |
| pMH76 | N-His_6_-CofC- p.M91A from M*. rhizoxinica* | This study |
| pMH77 | N-His_6_-CofC- M91L from M*. rhizoxinica* | This study |
| pMH80 | N-His_6_-CofC-G169S;T152H from *M. smegmatis* | This study |
| pMH81 | N-His_6_-CofC-G169S;L98C;T152H from *M. smegmatis* | This study |
| pMH82 | N-His_6_-CofC-T152H from *M. smegmatis* | This study |
| pMH89 | N-His_6_-CofD from *Ca.* E. factor | This study |
| pMH90 | N-His_6_-CofD from Archaeon_GBE54128 | This study |
| pMH91 | N-His_6_-CofD from *Ca.* H. Archaeon | This study |
| pFS02 | N-His_6_-CofD from *M. rhizoxinica* | This study |
| pFS03 | N-His_6_-CofC from *M. rhizoxinica* | Braga et al. 2019 (2) |
| pFS04 | N-His_6_-CofD from *M. jannaschii* | Braga et al. 2019 (2) |

References

1. Braga, D., Hasan, M., Kröber, T., Last, D., and Lackner, G. (2020) Redox coenzyme F_420_ biosynthesis in Thermomicrobia involves reduction by stand-alone nitroreductase superfamily enzymes. *Appl Environ Microbiol* **86**

2. Braga, D., Last, D., Hasan, M., Guo, H., Leichnitz, D., Uzum, Z., Richter, I., Schalk, F., Beemelmanns, C., Hertweck, C., and Lackner, G. (2019) Metabolic pathway rerouting in *Paraburkholderia rhizoxinica* evolved long-overlooked derivatives of coenzyme F_420_. *ACS Chem Biol* **14**, 2088-2094
