# Supplementary material for: Diversification by CofC and Control by CofD Govern Biosynthesis and Evolution of Coenzyme F420 and Its Derivative 3PG-F420
Source: mBio. 2022 Jan 18;13(1):e03501-21. doi: 10.1128/mbio.03501-21 (PMC8764529; doi:10.1128/mbio.03501-21)
Supplement: TABLE S4 [file mbio.03501-21-st004.docx]

**Table S4. Statistics of the X-ray diffraction data collection and structure refinement.**

| **Data collection** |  |
| --- | --- |
| Detector | Pilatus 3S 2M |
| Radiation source | BESSY, beamline 14.1 |
| Wavelength | 0.9184 Å |
| Resolution range (last shell) | 50 – 2.35 Å  (2.49 – 2.35 Å) |
| Space group  *a*-axis / *c*-axis | P3_2_21  57.09 Å 228.96 Å |
| Number of independent reflections (last shell) | 18963 (2983) |
| Completeness (last shell) | 99.9% (99.4%) |
| Redundancy (last shell) | 10.1 (10.6) |
| I/σ(I) (last shell) | 5.4 (1.3) |
| R_meas_ (last shell) | 0.489 (1.870) |
| cc_1/2_ (last shell) | 0.975 (0.511) |
| Wilson *B*-factor | 29.9 Å^2^ |
| **Refinement** |  |
| R_cryst_ / R_free_ (test data set with 5 % of all data) | 0.187 / 0.238 |
| Number of non-hydrogen atoms of CofC / ligands / water | 2951 / 75 / 194 |
| **Average isotropic *B*-factors (including the TLS contribution)** |  |
| Protein main chain / side chain | 27.2 / 30.2 Å^2^ |
| Water | 27.6 / 18.3 Å^2^ |
| **R.m.s. deviations from ideal geometry** |  |
| Bond lengths | 0.012 Å |
| Bond angles | 1.8° |
| Torsion angles | 7.3° |
| Molprobity score/percentile | 3.04 /100^th^ |
| Protein data bank entry | 7P97 |
